# HAV in fresh vegetables: a hidden health risk in district Mardan, Pakistan

**DOI:** 10.1186/2193-1801-3-675

**Published:** 2014-11-18

**Authors:** Waleed Khan, Muhammad Irshad, Gauhar Rehman, Anwar Hussain

**Affiliations:** Department of Botany, Shankar Campus, Abdul Wali Khan Univerity, Mardan, 23200 Khyber Pakhtunkhwa Pakistan; Department of Zoology, Shankar Campus, Abdul Wali Khan Univerity, Mardan, 23200 Khyber Pakhtunkhwa Pakistan

**Keywords:** Hepatitis A virus, Domestic sewage, Vegetables, PCR

## Abstract

Hepatitis A is an acute inflammation of the liver caused by the hepatitis A virus (HAV) in human. The path of entry of HAV to the bloodstream is the epithelium of the intestine. Liver inflammation occurs when HAV multiplies within the hepatocytes and Kupffer cells of the liver. HAV is mostly transmitted by contaminated water, fruits and vegetables. The purpose of the current study was to evaluate fresh vegetables raised on the fecal contaminated water for the detection of HAV by PCR method. Twenty nine samples were collected from 13 different locations of district Mardan and screened for the presence of HAV. Village Bajowro near Takht Bhai was the most contaminated site having HAV in all vegetables grown over there. Water samples collected from this area proved to be contaminated with HAV. It may be concluded that fecal contaminated water is unsafe for irrigation because of the health risk associated with such practices.

## Introduction

Viruses are the most familiar causes of food-borne infections in human population. They remain inert in or on contaminated food items which may transmit infection. Besides several groups of viruses responsible for the contamination of food items, HAV and gastroenteritis viruses are the major food-borne viral pathogens which infect via the gastrointestinal tract (Vaughan et al. 2013; Lee et al. 2013). Viruses responsible for infection via the gastrointestinal tract are expelled in vomit and may likewise be excreted in feces. Viral pathogen may contaminate foods either directly from infected people or through sewage pollution (Seymour and Appleton 2001). Untreated or inadequately treated sewage is one of the key factors contaminating food and water (Iritani et al. 2014). Fruits and vegetables that are consumed without cooking are particularly risky in spreading viral pathogens.

HAV may make its way to vegetables from sewage containing human feces (Bosch 1998; Rodríguez-Lázaro et al. 2012). HAV effectively resists detergents, acids (even pH 1), solvents and extreme temperatures (up to 60°C) (Wright 2013; Cappellozza et al. 2012). These characters make it suitable for survival in sewage of domestic origin. Its survival time may reach up to months even years in fresh and salt water (Sobsey et al. 1986).

Mardan is the second largest city of Khyber Pakhtunkhwa Pakistan, which occupies an area of 1,632 km^2^, located at 34°12'0 N 72°1'60E and an altitude of 283 metres (928 ft) (Khan et al. 2011). A project of sewage treatment plant (STP) with total cost of Rs. 51.89 million was started by the Mardan Development Authority with the collaboration of Asian Development Bank in 1980. The project was completed and its control was handed over to Tehsil Municipal Administration (TMA) on July 14, 1999 to manage its functioning (Dawn News 2012: Mardan sewage treatment project rendered useless From the Newspaper Updated Apr 18, 2012 12:08 am). However, the project never worked and the dream of cleaner Mardan did not come true. For the same reason, practice of dumping sewage is directly into canals and other freshwater bodies without any pre-treatment is continued. Water in the canals are predominantly contaminated with sewage which is used to irrigate agriculture fields. Situation become verse in areas where domestic sewage is directly used for the purpose or irrigation. Purpose of the current study was to investigate the presence of HAV in fresh vegetables grown on the field irrigated with fecal contaminated water in the target area.

## Material and methods

**Figure 1:**
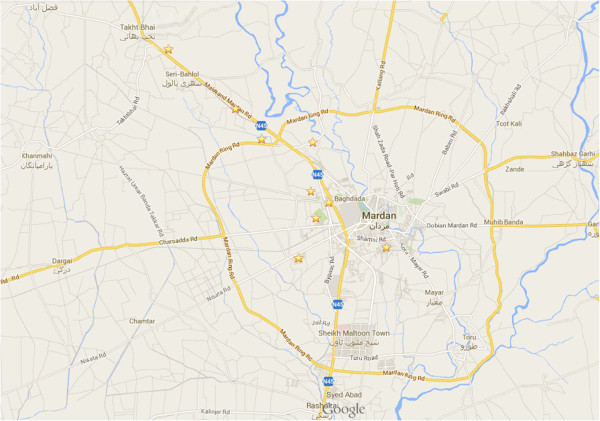
**Sampling sites are shown (starred) on the map of District Mardan Khyber Pakhtunkhwa, Pakistan.**

Different locations of district Mardan where agriculture fields are irrigated directly with domestic sewage or with the canals contaminated by domestic sewage, were selected for sampling. Sampling sites are shown on the map of district Mardan, Pakistan (Figure 1). Vegetables growing at the time of sampling (March-September, 2011) were collected and quickly frozen in liquid nitrogen. In lab, RNA extraction was accomplished by using Gene JET plant RNA purification kit (Fermentas, Germany) by following the manufacturer’s instructions. Seedlings fed with HAV positive serum, were used as positive control. Water samples were also processed for the extraction of viral RNA by using PowerWater^®^ RNA Isolation Kit (Carlsbad, CA USA). RNA was extracted from serum by using Favor prep viral nucleic acid extraction kit (Farvogen, Germany), according to its manufacturer’s protocol. Thirty μL of RNA samples were taken in properly labelled micro-centrifuge tubes. To the RNA samples, 30 μL of PCR Master Mix and 1.25 μL Reverse transcriptase (RevertAid™, Fermentas; 200 U μL^-1^) were also added. To this mixture, 100 pmol of antisense primer (Table 1) were added in each centrifuge tube and incubated at 42°C for 50 min (Croci et al. 2002). The reaction was stopped by incubating the reaction mixture at 95°C for 3 min. Thirty μL PCR Master Mix, 2.5 μL *Taq* polymerase (2.5 U) and 2.25 μL of sense primer (100 pmol) were added to RT-PCR product. The final volume of the reaction mixture was adjusted to 90 μL with nuclease free water. The cDNA was amplified via PCR under conditions described earlier (Croci et al. 2002). Twenty μL of each PCR product was mixed with 3 μL of 6X bromophenol blue (Fermentas) and were resolved on 1% agarose gel in a running buffer containing 0.2 μg/mL ethidium bromide. To determine size of amplified DNA fragments on the agarose gel, 3 μL of 100 bp DNA ladder (Fermentas) was used. Gel documentation system (BioDoc/It. 220 imaging system, S/N 51410–006, Cambridge, UK) was used to observe PCR products on the gel and to capture its image.

**Table 1:** **Sequence of sense and antisense primers used in PCR and RT-PCR**

S.No	Primers	Sequences	Reference
1.	Antisense primer	5′-CAGGGGCATTTAGGTTT-3′	Croci et al. 2002
2.	Sense primer	5′-CATATGTATGGTATCTCAACAA-3′	

## Results

During our survey, we found that domestic sewage was running directly to canals irrigating different areas of Mardan. In some places, such as Bajawro kalay and Guli Bagh, fields were irrigated directly with domestic sewage containing human feces. The areas were also best for raising vegetables among different locations of district Mardan. According to the local community, different vegetables including tomato, peppermint, coriander, radish, turnip, cabbage, potato, okra, chili, apple gourd and bottle gourd etc.

The sampling sites were selected on the basis of fertility of the agriculture fields mainly cultivated with vegetables and bad quality of water used for irrigation purpose. Different vegetables were sampled from thirteen different locations of Mardan. Amplification of 416 bp DNA fragment with HAV specific primers served to reveal the presence of HAV in the extract of the vegetable under question. A clear band of the mentioned size in the positive control (extract from vegetables fed with HAV positive serum) validated the procedure of RNA isolation and PCR conditions. Location-wise list of vegetables along with results indicating the presence or absence of HAV in these vegetables is given in Table 2. The area of Sazuddin is located of Mardan city. By the time we visited the area for sampling, *Brassica oleraceae*, *Solanum lycopersicum* and *Luffa acutangula* were grown. Most of the vegetables were quiet healthy and fresh with no visible sign or symptoms of disease. No HAV threat could be confirmed by the current study, evidencing that vegetables grown in the area might be safe for human consumption.

**Table 2:** **Location-wise list of vegetables along with results indicating the presence or absence of HAV in these vegetables determined by RT-PCR**

S.No.	Vegetables	Location	HAV
1	Cauliflower	L1. Sazodin near Rehman cotton mill Takht Bhai.	-
2	Tomato		
3	Ridge gourd		
4	Brinjal	L2. Samar Bagh Takht Bhai	-
5	Ridge gourd		
6	Pumpkin		
7	Ridge gourd	L3. Bajawro kalay near Tablighi Markaz Takht Bhai	+
8	Pumpkin		
9	Mint		
10	Bitter gourd	L4. Chail, Takht Bhai	-
11	Pumpkin		
12	Bitter gourd	L5: Western Bypass near Research Form Malakand road Mardan	-
13	Pumpkin		
14	Pumpkin	L6: Sharmakhano bridge and Lodhi Abad Gujargharai, Mardan	-
15	Ridge gourd		
16	Ridge gourd	L7: Deuband colony Bypass road Muqam, Mardan	-
17	Okra		
18	Pumpkin	L8: Miangul kalay near, Bypass road, Mardan	-
19	Pepper		
20	Pepper	L9: Bachakalay, Mardan	-
21	Pumpkin		
22	Brinjal		
23	Ridge gourd	L10: Guli Bagh, Mardan	-
24	Okra		
25	Ridge gourd	L11: Meervas kalay Charsadda road Mardan	-
26	Pumpkin		
27	Ridge gourd	L12: Tauheed colony near Charsadda chowk, Mardan	-
28	Okra		
29	Cucumber	L13: Near Fazli Haq College Mardan	-

Contaminated water drains and field of Samar Bagh irrigated with feces loaded water supported the growth of fresh and healthy vegetables, *Solanum melongeana*, *L. acutangula* and *Cucurbeta pepo*. PCR results proved that the vegetables of the area were free of HAV and hence, may be recommended for human consumption.

**Figure 2:**
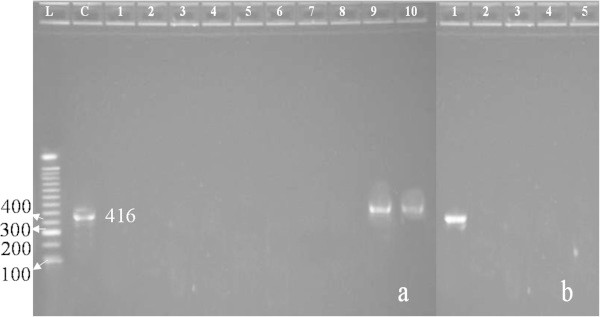
**Single PCR product (416 bp) in wells labelled as c (control), 9a (** ***L. acutangula*** **), 10a (** ***C. pepo*** **) and 1b (** ***M. piperita*** **), evidencing the presence of HAV in vegetables sampled from Bajowro kale near Takht Bhai.** Agarose gel shows RT-PCR results of different vegetable samples. No PCR product was detected in vegetables collected from Location 1 (1a, Cauliflower; 2a, tomato; 3a, Ridge-gourd ), Location 2 (4a, Brinjal; 5a, Ridge-gourd; 6a, Pumpkin), Location 4 (7a, Bittergourd; 8a, Pumpkin), Locaton 5 (2b, Bittergourd; 3b, Pumpkin), and Location 6 (4b, Pumpkin; 5b, Ridge-gourd).

Dumping of domestic sewage into drains and ultimately to the canal was documented in Bajawro kalay near Tableghi Markaz, Takht Bhai. This area was the most polluted among the sampling sites. At the time of sampling, *L. acutangula*, *C. pepo* and *Mentha piperita* were collected from the fields. A band (416 bp) was detected on agarose gel in all the vegetables collected from the site (Figure 2), evidencing the presence of HAV. Additionally, HAV was also present in water sampled from this area.

*Momordica charantia* and *C. pepo* were collected from fields of Chail, Takht Bhai. No band of DNA was detected in the PCR products using RNA extract from these vegetables, confirming the absence of HAV. Hence, the vegetables may be safe for human consumption.

People of Western bypass near Tobacco Research station, Malakand road Mardan are mostly illiterate, involved in agriculture by profession and green vegetables are grown all over the area. We sampled *M. charantia* and *C. pepo* from the area. The vegetables were free of HAV contamination as confirmed by the absence of PCR product on the gel.

*C. pepo* and *L. acutangula,* sampled from the fields in Sharmakhanon Bridge, Gujar Garhai. These vegetables were free from HAV and may be acceptable for human consumption. Similarly, *L. acutangula* and *Abelmoschus esculentus* grown in Deuband Muqam bypass road, Mardan were also free of HAV. No HAV RNA was detected in *C. pepo* and *Capsicum annuum* of Miangulkalay bypass barafkhana, Mardan.

Canal flowing through Bacha kalay, Mardan, located at a distance of 2.5 Km from Mardan city was also heavily polluted by human feces. However, vegetables grown in the area, *C. annuum*, *Cucurbeta pepo* and *S. melongeana*, were free of viral (HAV) contamination.

Guli bagh is an area located in the main city in close proximity to District Headquarter Hospital, Mardan. Canal passing through this area is heavily polluted with domestic sewage and hospital waste. Domestic sewage is also directly used for irrigating fields on which a number of different vegetables are grown. *A. esculentus* and *L. acutangula* were collected from this contaminated area. No HAV was confirmed by PCR.

The site of Sabzi Mandi Mirwas, Mardan, located at a distance of 1 Km from Mardan city, is moderately populated with 40 houses of the local population. *Cucurbeta pepo* and *L. acutangula* were collected from this area. No band corresponding to HAV RNA was detected on the gel indicating that the vegetables collected from this area were free from HAV contamination.

HAV contamination was not detected in the vegetables collected from Tauheed colony, Charsadda road, Mardan, which is located at a distance of 2 Km from Mardan city and are near Fazli Haq College, Mardan, about 3 Km from main city.

## Discussion

Vegetables from the most polluted area of district Mardan, where irrigation with fecal contaminated water is a common practice, were analyzed for HAV, a food born pathogen. Out of thirteen different locations, HAV was detected in the vegetables grown in Bajowro kalay only. We used RT-PCR based detection method for HAV detection. The method was previously used with success to determine the absorption of mammalian virus by vegetables (Seymour and Appleton 2001). Molecular biology techniques have been proposed for the sensitive and specific detection of some enteric viruses (Jean et al. 2001). The presence of HAV in three vegetables (*C. pepo*, *M. piperita* and *L. acutangula*) sampled from the area may be regarded as a hidden health risk to human population consuming such contaminated vegetables. The result of this study supported the idea that irrigation with fecal contaminated water may lead to HAV contamination of the crop. There are reports about the importance of HAV as a waterborne pathogen and the phenomenon is well recognized (Deboosere et al. 2012; Miura et al. 2013; Schultz and Myrmel 2013). Among the vegetables contaminated with HAV, *M. peperita* is consumed in its raw form and hence, may be a greater risk. Based on this postulation, other vegetables grown in such areas and consumed in raw form can be equally hazardous for humans. The initial survey has indicated the cultivation of tomato cucumber, onion, coriander, radish, carrot etc. on these fields which may magnify the intensity of the associated risk because of their consumption in raw form. Additionally, the HAV contaminated vegetables which are consumed in cooked form, may also contribute to the risk by communicating the virus to the HAV free vegetables and fruits consumed in raw form, through the use common kitchen utensils. Several HAV outbreaks due to consumption of berries and vegetables have been reported previously (Butot et al. 2007). Reports on contamination of vegetables from several parts of the world (Mukomolov et al. 2012; Hernández et al. 1997; Felix-Valenzuela et al. 2012) indicate the gravity of the situation.

It may be concluded that crop irrigated with sewage water may be screened not only for HAV, but also for other mammalian viruses, especially human pathogens of viral, bacterial, fungal and other origin. Based upon this study, the human consumption of vegetables grown on fields irrigated with fecal contaminated water is not recommended. Such ill practices need prompt attention of the government and civil society. Society mobilization against such practices is highly desired and the purpose may be achieved by educating people to understand this hidden health risk.
